# Three year survival among patients with aids-related Kaposi sarcoma treated with chemotherapy and combination antiretroviral therapy at Moi teaching and referral hospital, Kenya

**DOI:** 10.1186/s13027-019-0242-9

**Published:** 2019-09-10

**Authors:** Naftali Busakhala, Gabriel Kigen, Paul Waako, R. Matthew Strother, Fredrick Chite, Patrick Loehrer

**Affiliations:** 1 Department of Pharmacology and Toxicology, AMPATH Haemato-oncology institute, Moi University School of Medicine, P. O. Box 4606-30100, Eldoret, Kenya; 2 Department of Pharmacology and Therapeutics, Makerere College of Health Sciences, P. O. Box 3833, Kampala, Uganda; 3 Oncology Department, Canterbury District Health Board and Department of Medicine University of Otago, Christchurch, New Zealand; 4 Field Director of AMPATH Oncology and Haematology, P.O. Box 4606-30100, Eldoret, Kenya; 5 Indiana University Simon Cancer Center, 535 Barnhill Dr, Indianapolis, IN46202 USA

**Keywords:** AIDS-related Kaposi sarcoma, Survival, Kenya

## Abstract

**Background:**

AIDS-related Kaposi sarcoma (AIDS-KS), a common malignancy in Kenya is associated with high morbidity and mortality. AIDS-KS is treated using bleomycin and vincristine (BV) plus or minus doxorubicin in most low resource settings, with response rates ranging from 24.8 to 87%. Survival in low resource settings has not been well documented. We report the three-year survival in a cohort of seventy patients referred to Moi Teaching and Referral Hospital (MTRH).

**Methods:**

Study participants are part of a randomized phase IIA trial on the use of gemcitabine compared to bleomycin plus vincristine for the treatment of Kaposi sarcoma after combination antiretroviral therapy (cART) in Western Kenya. All patients were followed for three years in MTRH. Survival was determined by three monthly physical examination and analysed using Kaplan-Meier method, while possible determinants of survival such as baseline characteristics, type of chemotherapy, initial CD4 counts, age at enrolment, gender and early response to chemotherapy were analysed using univariate and multivariate Cox regression.

**Results:**

Participants were aged between 19 and 70 years with 56% being male. The median CD4 count was 224 cells/μl, median duration of HIV diagnosis was 12.0 months and median duration of KS lesions after histology diagnosis before initiating chemotherapy was 4.8 weeks. At three years, 60 (85.7%) patients were alive. Six of those who died were under treatment with BV while four with gemcitabine. There was no difference in the probability of survival between the patients on either treatment arm (HR = 0.573 [95% C. I 0.143, 2.292; *p* = 0.4311]). Additionally, the hazard ratio (HR) for response after six weeks, age at enrolment and gender indicated that they were not significant determinants of survival. Patients with normal CD4 cell counts (> = 500/μl), had a HR of 0.401(0.05,3.23; *p* = 0.391), suggesting better survival.

**Conclusions:**

Patients with AIDS-KS treated with combined antiretroviral drugs had excellent three-year survival regardless of whether they were treated with BV or gemcitabine as first line therapy. An initial CD4 cell count of > = 500/μl appeared to improve survival while gender, age and early response to chemotherapy were not predictors of survival after three years.

**Trial registration:**

Number PACTR201510001.

**Electronic supplementary material:**

The online version of this article (10.1186/s13027-019-0242-9) contains supplementary material, which is available to authorized users.

## Introduction

AIDS-related Kaposi sarcoma (AIDS-KS) is one of the most common malignancies in Kenya and sub-Saharan Africa and is associated with high morbidity and mortality [1–3]. AIDS-KS is caused by human immune deficiency virus (HIV) co-infection with human herpes virus type 8 (HHV-8) [4]. Histological examination of KS specimens shows proliferating spindle cells, inflammatory infiltrates and extensive angiogenesis, with spindle cells containing the latency-associated nuclear antigen [5, 6]. Both HHV-8 and HIV infection are highly prevalent in East Africa, thus causing the AIDS-KS epidemic in the region [7–9]. Usually, it develops among HIV patients who do not receive combination antiretroviral therapy (cART), although some cases have been reported in patients under treatment [2, 3, 10]. Men and women are almost equally affected due to the heterosexual nature of HIV transmission in Kenya [3].

Most patients present with extensive tumour combined with low CD4 counts and systemic illness, or poor risk disease based on the AIDS Clinical Trials Group (ACTG) staging criteria [11, 12]. Good risk AIDS-KS may resolve with cART alone, but poor risk AIDS-KS requires chemotherapy. The chemotherapeutic agent of choice in resourced setting is pegylated liposomal doxorubicin [13–16]. Most hospitals in low resource countries lack liposomal anthracyclines, and therefore AIDS-KS is treated using bleomycin and vincristine (BV) plus or minus doxorubicin. Some hospitals in these countries use single agent vincristine as first-line therapy while gemcitabine and paclitaxel are used as second-line therapy, where available [17, 18]. Response rates to BV range from 24.8 to 87% depending on the stage at diagnosis, performance status and adherence to chemotherapy [14, 17]. Knowledge on survival rates in an institution and how it compares to other centres is therefore important to inform both prognosis and treatment in order to improve care.

Data on the survival rates in Kenya are currently limited. A study conducted at Kenyatta National Hospital (KNH), the largest referral hospital in Kenya, reported that only 20% of patients treated for AIDS-KS continue to undergo monitoring visits after the tenth week from the beginning. Seventy-three percent got lost to follow-up, while 7% died [19]. The duration of response among AIDS-KS patients can be very short. For instance, in a trial comparing liposomal daunorubicin versus BV plus doxorubicin (ABV) conducted in USA, the median duration of response was only 3.8 months with a median survival time about one year [13]. In South Africa, the 2-year survival was reported to be 79%, while in Zambia, it was 57% [20, 21]. In Guinea, West Africa, 1-year survival was only 25% while in USA, 4-year survival was 63% [20–23]. Unpublished data from routine clinical care at Moi Teaching and Referral Hospital (MTRH) suggest much longer survival than both KNH and the USA study [24, 25]. These variable survival rates led us to ascertain the 3-year survival rates in our study, based on data from an ongoing randomized phase IIA trial that compares the efficacy of gemcitabine and bleomycin plus vincristine among 70 patients with HIV-related Kaposi sarcoma [26].

## Methods

### Study settings

The study was conducted at MTRH, a hospital located 320 km west of the capital city, Nairobi. It offers health services to the residents of Western Kenya, Eastern Uganda and Southern Sudan, a population of about 24 million people [24]. It also hosts a consortium of health care providers referred to as the Academic Model Providing Access to Healthcare (AMPATH) which has enrolled over 220,000 patients with HIV infection, of these 97,000 are treated with cART; one of the highest numbers of patients with HIV in Africa under a single provider [25]. The institutions that form AMPATH are MTRH, Moi University School of Medicine and a consortium of North American and European Universities led by Indiana University. The hospital has a dedicated cancer centre with outreach clinics in the neighbouring towns of Kitale, Webuye, Busia and Chulaimbo. It also has an AIDS Clinical Trials (ACTG) and AIDS Malignancy Consortium (AMC) Clinical Research Site which has been enrolling patients since 2006 [25].

Early AIDS-KS patients are usually treated using cART alone, or with addition of chemotherapy upon disease progression, whereas advanced AIDS-KS patients are administered both cART and chemotherapy up front. The combination of bleomycin (10 IU/m^2^) with vincristine (1.4 mg/m^2^) administered intravenously every 2 weeks, is used as first line chemotherapy. Gemcitabine administered intravenously at 1000 mg/m^2^ every 2 weeks is used as second-line chemotherapy.

### Study design and participants

Starting from May 2014, 70 patients with AIDS-KS were enrolled from a randomized phase IIA trial comparing the efficacy of gemcitabine to BV. After determination of efficacy at six weeks, patients continued to be treated and followed up for three years. Participants were examined every three months. Survival rates were assessed by analysing the parameters that affect the overall health of a patient, and evaluating how each may have contributed to the probability of survival over the follow-up period. These included the baseline characteristics, type of chemotherapy regimen, initial CD4 counts done at the initiation of chemotherapy, age at enrolment and gender. We also investigated whether an early response to chemotherapy influenced survival by comparing the survival curves of the patients who responded well after the first three cycles (six weeks) to those who did not, or required further cycles of chemotherapy.

### Inclusion criteria

The study participants were aged 18 years and above, HIV-positive diagnosed by two approved rapid test kits namely Alere determine™ (Alere, Mitsudo, Japan) and Uni-Gold™ (Trinity Biotech, Ireland) in accordance with the Kenyan national guidelines for diagnosis of HIV [27]. In addition, the participants had to be under cART treatment for at least 8 weeks prior to enrolment to avoid confusing immune reconstitution syndrome of AIDS-KS with progressive disease. Due to unreliable history from study participants, it is difficult to tell whether anyone had developed KS while on cART. The first-line cART regimen commonly comprised of tenofovir or zidovudine combined with lamivudine and efavirenz or nevirapine. In this study, six participants were treated with lopinavir/ritonavir instead of efavirenz. Lamivudine was retained in all regimens as recommended by the national guidelines. Skin biopsies were done and read by the study pathologist at MTRH. The histology slides and paraffin embedded blocks were sent to University of California, San Francisco for second reading and staining for latency-associated nuclear antigen (LANA-1) to confirm the diagnosis. Laboratory parameters had to be normal for participants, including white cell count, haemoglobin, platelet count, liver functions, kidney functions and performance status using the Eastern Co-operative Oncology Group (ECOG) score of ≤2. Exclusion criteria included previous treatment with chemotherapy or radiotherapy as well as unwillingness to use contraception, being pregnant or breast feeding. Co-morbidities such as tuberculosis, pneumonia, lymphoma, hypertension and diabetes were actively investigated and excluded prior to enrolment. The study protocol was approved by the Institutional review board (IREC NO 000490) and the national expert committee on clinical trials at the Ministry of health (PPB/ECCT/11/10/01/2013). All participants signed a voluntary informed consent.

Complete blood counts (including neutrophils, platelet, and haemoglobin), liver and renal function tests were done at every cycle prior to treatment. Chemotherapy was delayed for patients with absolute neutrophil count less than 1000 × 10^3^/μl or absolute platelet count below 100 × 10^3^/μl. Filgrastim 300mcg daily for 5 days was administered for neutropenia while platelet transfusion was given for severe thrombocytopenia. Nutritional counselling and support were provided according to World Health Organisation guidelines. The saturation of arterial oxygen as measured by a pulse oximeter and creatinine levels were normal throughout the study.

Participants were provided with supportive care both as day care and admission when necessary. The research assistants and a community navigator continuously counselled participants and their relatives. This was done after every two weeks while getting chemotherapy, and 3-monthly after completion of chemotherapy ensuring that there was no loss to follow-up during the study period.

Response was determined through bidirectional measurement of the index lesion using a Vernier calliper as well as monitoring the development of other lesions. Photographs of involved skin lesions were taken at every cycle by the same person and camera to avoid inter-observer variability. Complete response (CR) was defined according to the ACTG criteria as resolution of any detectable disease for at least 4 weeks including KS-associated oedema. We did not do repeat biopsies to confirm complete pathologic response due to resource constraints. However, this was validated by the absence of new lesions after long-term follow up. Partial response (PR) was defined as at least 50% reduction in the size of the index lesion and the absence of any new lesions lasting at least 4 weeks. Progressive disease (PD) was defined as at least 25% increase in the size of the index lesion or development of new lesions or worsening of tumour-associated oedema. Stable disease (SD) was defined as any disease not meeting criteria for CR, PR or PD. Relapse was defined as recurrence of lesions at least 4 weeks after achieving CR.

After the first three treatment cycles of either gemcitabine or BV under drug trial conditions, analysis for efficacy of the study drugs was done and results published [26]. Study participants were transitioned to the routine oncology clinic and followed up for survival under standard care. During follow-up, patients with recurrent or progressive disease were administered second-line or third-line chemotherapy. Second-line chemotherapy comprised of gemcitabine for those who had failed BV, or BV for those who had failed gemcitabine while paclitaxel or liposomal doxorubicin was given as third-line chemotherapy.

### Statistical analysis

Data analysis was performed using R Core Team (2016). Categorical variables were summarized as frequencies and the corresponding percentages while continuous variables were summarized as median and interquartile range (IQR). Survival was analysed according to Kaplan and Meier method, while associated factors were analysed using univariate and multivariate Cox regression.

## Results

### Characteristics of the study participants

The characteristics of patients in both arms of the study were comparable (Table 1). Participants were aged between 19 and 70 years, with a median age of 35.6 (IQR: 30.6, 41.6) years, and 56% being male. The median CD4 cell count at enrolment was 224 (IQR: 107.0, 360.5) cells/μl (with a wide range of 4.0–824.0 cells/μl) which was similar in both groups. The median duration of HIV treatment was 12.0 (IQR: 6.0, 53.5) months with a range of 1.0–204.0, and the median duration of documented KS from histology diagnosis to initiating chemotherapy was 4.8 (IQR: 3.7, 8.8) weeks (range 2.0–157.9). Ninety-seven percent of participants had generalised KS (stage 1).

**Table 1:** Demographics and clinical characteristics of study participants at enrolment

	GEM (*n* = 36)	BV (*n* = 34)	Total (*n* = 70)	*p*-value
Males	16 (44.4%)	24 (70.6%)	40 (57.1%)	0.087
Age [years] (Median, IQR)	35.3 (30.0, 40.5)	36.9 (31.0, 44.1)	35.6 (30.6, 41.6)	0.565
Stage:				
0	1 (2.8%)	1 (2.9%)	2 (2.9%)	1.000
1	35 (97.2%)	33 (97.1%)	68 (97.1%)	
ECOG PS:				
0	19 (52.8%)	14 (41.2%)	33 (47.1%)	0.286
1	17 (47.2%)	17 (50.0%)	34 (48.6%)	
2	0 (0.0%)	2 (5.9%)	2 (2.9%)	
3	0 (0.0%)	1 (2.9%)	1 (1.4%)	
CD4-cells/μL (Median, IQR)	225.0 (108.5, 310.0)	222.0 (63.8, 431.0)	224.0 (107.0, 360.5)	0.914
BSA [m^2^] (Median, IQR)	1.57 (1.50, 1.70)	1.67 (1.58, 1.79)	1.60 (1.50, 1.74)	0.0872
Duration of HIV diagnosis [months] (Median, IQR)	14.5 (4.8, 66.8)	12.0 (6.3, 38.0)	12.0 (6.0, 53.5)	0.722
Duration of AIDS-KS confirmed diagnosis [weeks] (Median, IQR)	5.4 (3.9, 8.8)	4.3 (3.0, 7.9)	4.8 (3.7, 8.6)	0.300

Key: *BV* Bleomycin plus Vincristine, *GEM* Gemcitabine, *ECOG PS* Eastern Cooperative Oncology Group Performance Status, 67/70 Patients had good performance status. *BSA* Body Surface Area. Duration of KS from histology diagnosis to starting chemotherapy. Stage 1; Advanced KS, 68/70 patients had stage 1 KS according to ACTG criteria

### Survival rates after 3 years

The survival rate at 3 years was remarkable, with sixty out of 70 (85.7%) recruited patients having survived and no loss to follow-up. The mean number of chemotherapy cycles received by both groups was six, with the range for gemcitabine being 3–13 cycles and 3–11 for BV. Moreover, six patients treated with BV and four patients on gemcitabine received second-line or third-line chemotherapy due to recurrent or progressive disease. Time to recurrence or progression ranged from 4 weeks to 96 weeks. All these 10 patients died during follow-up.

**Fig. 1:**
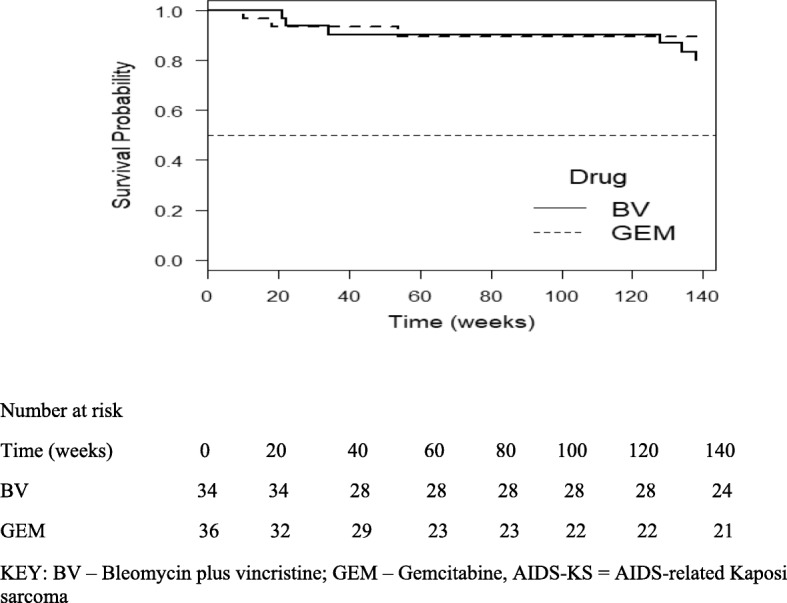
Overall Survival after 3 years in patients with AIDS-KS at MTRH treated with chemotherapy (HR = 0.573 [95% C. I 0.143, 2.292; *p* = 0.4311])

Kaplan-Meier survival curves of the participants on both study arms over the study period, show a similar pattern (the probability of survival at 3-years for both study arms were approximately 0.9, HR = 0.573 (95% C. I 0.143, 2.292; *p* = 0.4311) (Fig. 1). This result suggests that there was no difference in the probability of survival between the patients who were given BV or gemcitabine.

**Fig. 2:**
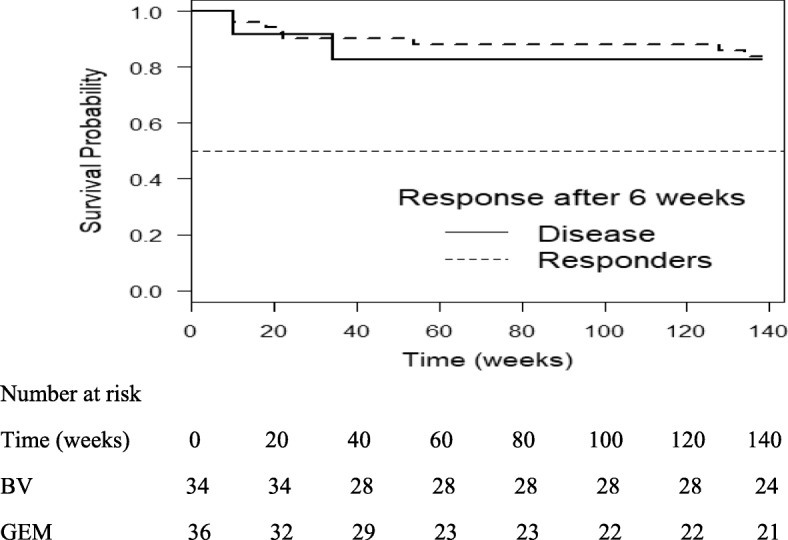
Three-year survival curves in patients who responded to chemotherapy after the first 6 weeks and those who had persistent disease (HR 1.212 (0.257, 5.716); *p* = 0.8018])

Figure 2 shows the survival curves of patients who responded to gemcitabine or BV after the first three cycles (6 weeks) and those who responded after more than 3 cycles. The probability of survival at 3 years for participants who responded after 3 cycles of chemotherapy and those who responded after more than 3 cycles of chemotherapy was 0.9.

### Influence of CD4 counts, response, age and gender on survival rate

Table 2 shows that in patients with normal CD4 cell counts (> = 500/μl) at the time of initiating chemotherapy, the HR was 0.401(0.05–3.23), *p* value 0.391, which means the hazard of death was reduced by 60% although it is not statistically significant. Both the unadjusted and adjusted Hazard Ratios for response after six weeks, age at enrolment and gender were not statistically significant determinants of survival as shown in Table 2.

**Table 2:** Hazard Ratios of identified determinants of 3-year survival

	Unadjusted HR (95% CI)	Unadj. p-value	Adjusted HR (95% CI)	Adj. p-value
Response after 6 weeks	0.961(0.208,4.45)	0.96	1.212(0.257,5.716)	0.808
Age at Enrollment (per 1-year increase)	0.944(0.867,1.027)	0.182	0.937(0.86,1.021)	0.136
Male gender	1.018(0.311,3.338)	0.976	1.294(0.387,4.332)	0.676
Initial CD4 = > 500/μl	0.483(0.062,3.777)	0.488	0.401(0.05,3.23)	0.391

Adjusted HR refers to adjustment for the other 3 determinants e.g. adjusted HR for Response after 6 weeks refers to the HR after adjusting for age, gender and initial CD4 cell count

In the unadjusted model for response after 6 weeks, the unadjusted hazard ratio was 0.961. The hazard of death is reduced by 3.9% in the responders compared to the disease group, however this difference is not statistically significant, HR = 0.961 (95% C. I 0.208, 4.45; *p* = 0.9596). In the adjusted model, the hazard for death is increased by 35.9% in responders compared to the disease group but again, the difference is not statistically significant, HR = 1.359 (95% CI 0.28, 6.592; *p* = 0.7035). In the unadjusted model for gender, the hazard of death is increased by 1.8% in males compared to females, however the difference is not statistically significant, HR = 1.018 (95% CI 0.311, 3.338; *p* = 0.976).

## Discussion

The overall 3-year survival of study participants was 85.7%. None of the known determinants of survival in patients with AIDS-KS analysed in this study including age, gender, type of chemotherapy received, CD4 cell counts at the start of chemotherapy and early response to chemotherapy were significantly associated with survival. In patients with normal CD4 cell counts (> = 500/μl) at the time of initiating chemotherapy, there was a trend towards better survival.

In our study, 57% of participants were males, 97% had stage 1 disease and 97% had good functional status (ECOG 0 and 1) consistent with the study population where males and females were almost equally affected by KS and most patients presented with advanced disease [28]. About 98% of participants had ECOG score of 0 or 1 despite advanced disease. This good functional status despite advanced disease appears unique to this study and may require more investigation because it may contribute to delayed presentation by patients for care. The mean age was 35.6 years which points to the potential of AIDS-KS reducing economic productivity by affecting a youthful population. The average initial CD4 count was 224/μl reflecting the advanced level of immune suppression in the study participants. The study participants were under treatment with cART for 4.8 to 66.8 (average 12) months, thus they either developed AIDS-KS or progressed despite anti-retroviral treatment but this is not easy to ascertain due to unreliable history from patients.

There was no difference in survival at 3 years among patients who received the local standard of care of BV compared to those who were administered the experimental drug (gemcitabine) because chemotherapy acts as adjuvant in AIDS-KS patients while main therapy is cART. Similar results were reported in a trial comparing cART alone, and cART plus ABV in South Africa. The overall survival in that study was 77% although response rates were higher in the cART plus ABV study arm [29]. Similar results were also reported in a phase III trial which compared liposomal daunorubicin with a combination of doxorubicin, bleomycin and vincristine (ABV) in which response rates, time to treatment failure and survival were similar [13]. In a study involving 254 consecutive patients with AIDS-KS in the United Kingdom where most of the study participants had early stage (T0) disease, only 22% received chemotherapy but the overall survival was 91% [30]. Some studies evaluating the effectiveness of gemcitabine in classic Kaposi sarcoma have reported response rates of 100% and long-term survival, even among patients refractory to liposomal doxorubicin [31]. It is important to note that some of the patients in this study continued to respond to gemcitabine after recurrence. This may be a therapeutic advantage of gemcitabine because unlike liposomal doxorubicin it has no life-time dose limit.

As previously reported in the results, among patients with normal CD4 cell counts (> = 500/μl) at the time of initiating chemotherapy, the HR was 0.401(0.05,3.23), *p* value 0.391, which means the hazard of death was reduced by 60% but this was not statistically significant. A retrospective study conducted in Uganda to evaluate the validity of ACTG criteria in sub-Saharan Africa did not find baseline CD4 to be a predictor of survival in AIDS-KS patients whether in the short run or after 2 years [32]. The study reported a poor survival rate of 57% at 2 years and unexpectedly did not find cART to be a predictor of survival probably because of incomplete access to records or use of low potency anti-retroviral drugs in Uganda from 1992 to 2007 when the study participants were treated. The other variables we analysed to determine relationship with survival included age, gender and early response to chemotherapy and none of them were statistically significant.

## Conclusion

Patients with AIDS-KS referred to MTRH taking anti-retroviral drugs had excellent 3-year survival regardless of whether they were treated with bleomycin plus vincristine or gemcitabine as first-line chemotherapy. Gender, age and early response to chemotherapy were not predictors of survival at 3 years. In patients with normal CD4 cell counts at the time of initiating chemotherapy, survival was improved but this was not statistically significant. In addition, results suggest that gemcitabine can be used as an alternative to bleomycin plus vincristine as first-line chemotherapy among patients with AIDS-KS on anti-retroviral therapy.

### Study strengths and limitations

Study strengths include comparison of an experimental group (gemcitabine) to standard of care group (BV) making it easy to make efficacy inferences. In addition, the prospective study design and randomisation also minimised recall and selection bias. Study limitations include a relatively small sample size and few deaths resulting in wide confidence intervals. The study was also done in a single centre with highly motivated participants thus limiting generalizability of the study findings to routine AIDS-KS patients.

## Additional file

Additional file 1: Patient data (XLSX 12 kb)

## Data Availability

All available data are included in the article text, and on the supplementary attached file (Additional file 1).

## Abbreviations

ABV: Doxorubicin [Adriamycin®], bleomycin and vincristine
ACTG: AIDS Clinical trials group
AIDS: Acquired immune deficiency syndrome
AMC: AIDS malignancy consortium
AMPATH: Academic model providing access to healthcare
BV: Bleomycin and vincristine
cART: Combination anti-retroviral therapy
CD4: Cluster of differentiation 4
ECOG: Eastern co-operative oncology group
HIV: Human immunodeficiency virus
HR: Hazard ratio
KS: Kaposi sarcoma
LANA-1: Latency-associated nuclear antigen
MTRH: Moi Teaching and Referral Hospital
NHIF: National Hospital Insurance Fund
